# Context‐dependent resistance of freshwater invertebrate communities to drying

**DOI:** 10.1002/ece3.2870

**Published:** 2017-03-31

**Authors:** Thibault Datry, Ross Vander Vorste, Edgar Goïtia, Nabor Moya, Melina Campero, Fabiola Rodriguez, Jose Zubieta, Thierry Oberdorff

**Affiliations:** ^1^ IRSTEAUR‐MALYcentre de Lyon‐VilleurbanneVILLEURBANNE CedexFrance; ^2^ UMR “BOREA” CNRS 7208/IRD 207/MNHN/UPMCUNICAENMuseum National d'Histoire NaturelleParis CedexFrance; ^3^ Unidad de Limnología y Recursos Acuáticos (ULRA)Universidad Mayor de San SimónCochabambaBolivia; ^4^ UNIBOL Quechua “Casimiro Huanca”ChimoréCochabambaBolivia; ^5^ UMR 5174 EDB, CNRS, UPSENFA ‐ Université Paul SabatierToulouseFrance; ^6^ Present address: Virginia Water Resources Research InstituteVirginia TechBlacksburgVAUSA

**Keywords:** climate change, coexistence, desiccation resistance, drying, neotropical streams and wetlands (bofedales)

## Abstract

More freshwater ecosystems are drying in response to global change thereby posing serious threat to freshwater biota and functions. The production of desiccation‐resistant forms is an important adaptation that helps maintain biodiversity in temporary freshwaters by buffering communities from drying, but its potential to mitigate the negative effects of drying in freshwater ecosystems could vary greatly across regions and ecosystem types. We explored this context dependency by quantifying the potential contribution of desiccation‐resistance forms to invertebrate community recovery across levels of regional drying prevalence (defined as the occurrence of drying events in freshwaters in a given region) and ecosystem types (lentic, lotic) in temporary neotropical freshwaters. We first predicted that regional drying prevalence influences the selection of species with desiccation‐resistant forms from the regional species pools and thus increases the ability of communities to recover from drying. Second, we predicted lentic freshwaters harbor higher proportions of species with desiccation‐resistant forms compared to lotic, in response to contrasted hydrologic connectivity. To test these predictions, we used natural experiments to quantify the contribution of desiccation‐resistant forms to benthic invertebrate community recovery in nine intermittent streams and six geographically isolated temporary wetlands from three Bolivian regions differing in drying prevalence. The contribution of desiccation‐resistant forms to community recovery was highest where regional drying prevalence was high, suggesting the species pool was adapted to regional disturbance regimes. The contribution of desiccation‐resistant forms to community recovery was lower in streams than in wetlands, emphasizing the importance of hydrologic connectivity and associated recolonization processes from in‐stream refuges to recovery in lotic systems. In all regions, the majority of functional traits were present in desiccation‐resistant taxa indicating this adaptation may help maintain ecosystem functions by buffering communities from the loss of functional traits. Accounting for regional context and hydrologic connectivity in community recovery processes following drying can help refine predictions of freshwater biodiversity response to global change.

## 1 Introduction

How biodiversity is produced and maintained in ecosystems is a pervasive goal of community ecology. When disturbances or strong environmental changes heavily alter species density, there are several niche‐ and dispersal‐based processes thought to promote population and community recovery (defined as maintenance of species composition following a disturbance, *sensu* Connell & Slatyer, 1977). One important niche‐based process is the production of persistent life‐history stages that buffer population growth from environmental fluctuations and help promote species coexistence. The production of persistent life‐history stages is recognized in virtually all communities, including coral reef fish (Chesson & Warner, 1981), lake zooplankton (Cáceres, 1997), river invertebrates (Stubbington & Datry, 2013), tropical trees (Runkle, 1989), and prairie grasses (Adler, HilleRisLambers, Kyriakidis, Guan, & Levine, 2006).

The recurrent and complete disappearance of surface water is global issue occurring naturally in many freshwater systems, whether they are lentic (e.g., Bie et al., 2012; Ruhí, Boix, Gascón, Sala, & Batzer, 2013; White, McHugh, & McIntosh, 2016) or lotic (e.g., Ledger, Edwards, Brown, Milner, & Woodward, 2011; Sponseller, Grimm, Boulton, & Sabo, 2010; van Vliet et al., 2013). However, the occurrence of drying events is becoming exacerbated by global change and shifts from permanent to intermittent water regimes in many regions are being observed (Gleick, 2003; Smol & Douglas, 2007) or predicted (Datry et al., 2014; Döll & Schmied, 2012; Pyne & Poff, 2017). This is particularly true in the dry regions of South America, and particularly in Bolivia (Beklioğlu et al., 2016; Gudynas, 2016). For example, during the recent El Niño period (2015–2016), Lake Poopó, Bolivia's second largest waterbody (after Lake Titicaca), dried completely for the first time ( http://news.nationalgeographic.com/2016/01/160121-lake-poopo-bolivia-dried-out-el-nino-climate-change-water/, Beklioğlu et al., 2016; Gudynas, 2016).

In intermittent streams and temporary wetlands, community recovery upon rewetting generally occurs within a few weeks (e.g., Datry et al., 2014; Leigh et al., 2016; Ruhí et al., 2013; Vander Vorste, Corti, Sagouis, & Datry, 2016). The production of desiccation‐resistant forms such as cysts, eggs, and dormant larva can partly explain this recovery (Datry, Moya, Zubieta, & Oberdorff, 2016; Larned, Datry, & Robinson, 2007; Tronstad, Tronstad, & Benke, 2005). For example, chironomids from the subfamilies Orthocladiinae and Chironominae (Order: Diptera)—a taxonomically and numerically dominant taxa in many streams and ponds—can survive several months under dry conditions (Larned et al., 2007; Tronstad et al., 2005). However, the contribution of this “seedbank” to community recovery is highly variable (e.g., Larned et al., 2007; Stubbington & Datry, 2013; Tronstad et al., 2005). For example, 50% of the species comprising stream communities of an intermittent stream in France were also found in sediments that had been dry for 5–320 days (Datry, Corti, & Philippe, 2012), suggesting a strong contribution of resistance forms to recovery; whereas, this contribution appeared nonexistent in other intermittent systems in France (Vander Vorste, Malard, & Datry, 2016) and in the USA (Stanley, Buschman, Boulton, Grimm, & Fisher, 1994). Therefore, the extent to which desiccation‐resistant forms contribute to community recovery in freshwater ecosystems must be studied across regions and ecosystem types before predicting the effects of increasing drying occurrence resulting from global change on freshwater biodiversity.

**Figure 1:**
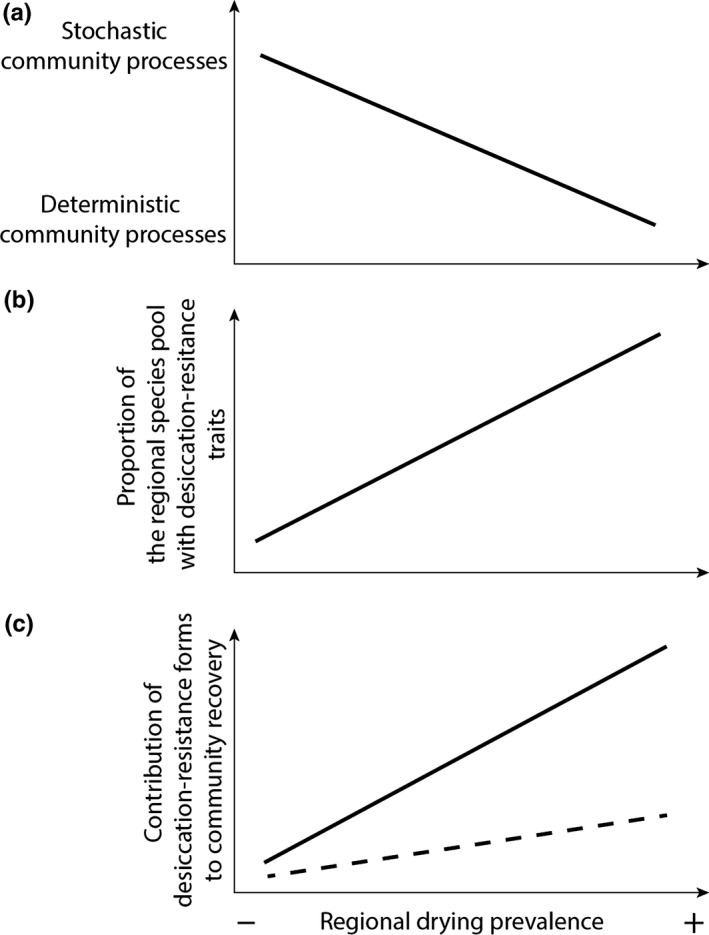
Predicted patterns of (a) the relative contributions of stochastic and deterministic community assembly processes; (b) the proportion of species in the regional species pool with desiccation‐resistant forms; and (c) the contribution of desiccation‐resistant forms to community recovery along a gradient of increased regional drying prevalence. Continuous lines: lotic (wetlands) and broken lines: lentic (streams) ecosystems. Differences in slopes are due to contrasted hydrologic connectivity between streams and wetlands

Context dependency in the contribution of desiccation‐resistant forms to community recovery in freshwaters could be driven by drying prevalence and hydrologic connectivity. First, regional drying prevalence (defined as the occurrence of drying events in freshwaters in a given region) is likely alter the contribution of resistance forms through changes in community composition. Following theory, in ecosystems prone to harsh environmental conditions, deterministic niche selection filters out species from regional pools unable to tolerate such conditions; conversely, stochastic processes such as ecological drift are thought to be important in more benign conditions (Chase, 2007; Ruppert et al., 2015; Vellend, 2010). Therefore, in regions where drying occurs in most aquatic systems, niche selection should retain species with desiccation‐resistant traits (Figure 1a,b). Conversely, in regions where drying is rare, only a few species should have desiccation‐resistant traits (Figure 1a,b). Second, lotic and lentic freshwater ecosystems differ in terms of hydrologic connectivity, which should in turn influence the contribution of desiccation‐resistance forms to community recovery. While aquatic habitats in wetlands are often hydrologically disconnected from each other during both dry and wet phases, those in intermittent streams are occasionally connected to each other by a directional flux of water, solutes, and organisms (e.g., Bogan, Boersma, & Lytle, 2013; Datry, Lamouroux, Thivin, Descloux, & Baudoin, 2015; Fagan, 2002). Water flow can indeed favor the colonization of rewetted sites by aquatic organisms originating from upstream or downstream perennial habitats and therefore reduce the selection of desiccation‐resistant traits. Passive or active recolonization from upstream/downstream refuges is often assumed to explain community recovery in streams (Bogan et al., 2013; Davey & Kelly, 2007; Vander Vorste, Malard, et al., 2016). Accordingly, at a similar level of regional drying prevalence, the contribution of desiccation‐resistant forms to community recovery should be higher in lentic compared to lotic ecosystems, assuming that species with aerial dispersal modes do not fully compensate for the decreased hydrologic connectivity in lentic ecosystems (Brock, Nielsen, Shiel, Green, & Langley, 2003; Bie et al., 2012).

Here, we tested whether the potential contribution of desiccation‐resistant forms differs consistently with regional drying prevalence and between lotic and lentic freshwaters. We quantified this contribution for invertebrate communities in nine intermittent streams and six geographically isolated temporary wetlands from three Bolivian regions with varying regional drying prevalence. We first predicted the contribution of desiccation‐resistant to community recovery to increase with the regional drying prevalence, due to a predominant selection of species with desiccation‐resistant forms from the regional species pool (Figure 1c). We second predicted this contribution to be higher in wetlands than in streams, at similar levels of regional drying prevalence, due to lower hydrologic connectivity and subsequently less passive or active recolonization potential from distant sources of colonists (Figure 1c). By identifying factors that lead to context dependency in the contribution of desiccation‐resistant forms, our results will improve predictions of the response of freshwater fauna, which are experiencing among the strongest declines in biodiversity of any faunal group (Dudgeon et al., 2006; Jenkins, 2003), to increased drying due to global change.

## 2 Method

### 2.1 Studied regions

**Figure 2:**
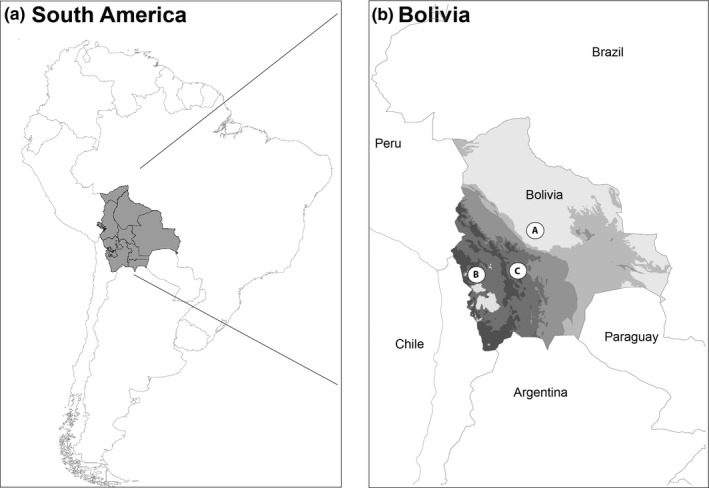
Map showing (a) Bolivia in South America and (b) the three studied regions. A. low drying prevalence, B. medium drying prevalence, C. high drying prevalence. Grayscale represent altitudes

Following a “gradient” approach, three regions of Bolivia with increasing regional drying prevalence affecting freshwater ecosystems were considered (Figure 2, Table 1). While it was impossible to replicate entire catchments due to ruggedness and accessibility of these areas and because catchments would differ in other aspects (e.g., level of human impacts, duration of drying periods), this design allowed us, however, to make inferences about the importance of drying prevalence and hydrologic connectivity on the contribution of desiccation‐resistant forms. In each region, drying prevalence was assessed quantitatively by i. annual water deficit (annual rainfall—evapotranspiration, Table 1) and ii. channel length (streams) or surface area (wetlands) without water during the dry periods.

**Table 1:** Environmental characteristics of the three studied areas used to define categories of drying prevalence From Datry et al., 2015; Moya et al., 2011; Navarro & Maldonado, 2002; Vicente–Serrano et al., 2015). Values represent averages across sampling sites

Region	Climate	Altitudinal range (m a.s.l.)	Annual rainfall (mm)	Annual temperature (°C)	Annual evapotranspiration (ET, mm)	Annual water deficit (Rainfall/ET)	Source of flow	% river network intermittent
Amazonian Piedmont	Tropical	220	1513.8	24–26	1779.4	–258.7	Groundwater/runoff	<5
Altiplano	Semi Arid	3,819–4,496	463.1	4–6	1546.0	–916.9	Glacier	20–50
Dry Central Valleys	Arid	2,280–3,286	568.8	10–25	1918.2	–1349.4	Runoff	90–95

The Amazonian Piedmont region of the Andes is a tropical and highly forested area located in the Bolivian Amazon catchment (Moya, Tomanova, & Oberdorff, 2007; Tedesco et al., 2007; Datry et al., 2016; Figure 2, Table 1). This region has an average altitude of 220 m a.s.l., and annual precipitation varies between 1,200 and 6,500 mm, with mean annual temperature between 24 and 26°C (Navarro & Maldonado, 2002). We defined this region as having a low regional drying prevalence (LOW) because of its low annual water deficit (Table 1) and because streams are fed by runoff and groundwater inputs allowing 90 to 95% of the river network to flow perennially (Tedesco et al., 2007). No geographically isolated wetlands are reported in this region. However, local hydrogeological conditions create small intermittent headwater streams that dry from April to September (Datry et al., 2016).

The Sajama region located in the Altiplano has an average altitude of 4500 m a.s.l., with annual precipitation between 160 and 490 mm and a mean annual temperature of 5.5°C (Navarro & Maldonado, 2002; Jacobsen & Marín, 2008; Moya, Gibon, Oberdorff, Rosales, & Domínguez, 2009; Figure 2, Table 1). The presence of many glaciers sustains relatively high flow permanence in streams and wetlands during the dry season, and about 50% of the freshwaters are intermittent (Datry et al., 2016; Moya et al., 2009). This region was defined as having a medium regional drying prevalence (MED) because there is a moderate annual water deficit (Table 1).

The dry central valleys of Cochabamba are located in an arid plateau ranging from 2,500 to 3,800 m a.s.l. (Navarro & Maldonado, 2002; Moya et al., 2011). Annual precipitation varies between 210 and 650 mm, with mean annual temperature between 10 and 25°C (Navarro & Maldonado, 2002). A severe annual water deficit (Table 1) in this region causes more than 80% of the river network to dry along with a large proportion of the wetlands during the dry season (Navarro & Maldonado, 2002; Moya et al., 2011, Figure 2, Table 1). Therefore, this region was defined as having a high regional drying prevalence (HIGH).

### 2.2 Studied streams and wetlands

In each region, we selected three intermittent streams and three temporary wetlands, except for the Amazonian Piedmont where no wetlands were found. Selection was made with effort to minimize differences in length, width and substrate characteristics for streams and in surface area and substrate characteristics for wetlands (Appendix S1). We also controlled for drying regime by selecting sites that dry continuously and predictably during the austral winter (April–September) for approximately the same duration (4–6 months, Moya et al., 2009; Datry et al., 2016). This was confirmed by visual observations made by local people we met during field sampling trips. Sites were fairly evenly distributed longitudinally along streams, which were on average ~15 km long and 1–15 m wide without major tributaries (Appendix S1) to reduce within‐stream variability in environmental conditions. Wetland sites consisted of individual pools that were fairly evenly distributed across broader wetland areas of 45,000 m^2^ on average (Appendix S1) to reduce within‐wetland variability in environmental conditions.

### 2.3 Experimental design

Dry sediments were collected from 3 to 6 sites in each stream and wetland in July and August 2014, 3 months after the start of the dry period. Sediments were then artificially inundated in the laboratory for 2 weeks (see below). Sites were selected evenly along the longitudinal gradient in streams and across the extent of surface area in wetlands. In February and March 2015, approximately 2 months after the return of surface water, invertebrate communities were sampled from the same sites (see below). Two months was previously shown to be sufficient to allow the recovery of invertebrate communities in many regions (Temperate, Datry, 2012; Mediterranean, Bonada, Rieradevall, Prat, & Resh, 2006; Arid, Bogan et al., 2013; Tropical; Leigh et al., 2016), including the ones studied here (Datry et al., 2016; Moya et al., 2009). Pairwise comparisons of samples collected at both periods (dry and wet) were then carried out to explore context dependency in the contribution of desiccation‐resistant forms (see below).

#### 2.3.1 Dry sediment collection and inundation

At each site, 1 L of dry sediments was collected from three haphazard locations in three different riffles (streams) or ponds (wetlands) and then pooled together to form a composite sample of 3L. Sediments were excavated from a 0.1‐m^2^ surface area with a shovel and to a depth of 10 cm, placed into a 5‐L container and brought back to the laboratory within 5 hr. In the laboratory, each container was inundated with 2 L of filtered (250‐μm), nonchlorinated tap water, continuously aerated using airstones, and fitted with 1‐mm mesh lids to retain emerging insects and prevent colonization, following a widely used protocol (e.g., Datry et al., 2012; Larned et al., 2007; Storey & Quinn, 2013). In order to mimic normal inundation conditions of local temperature and moisture content, dry sediment samples from the MED and HIGH regions were inundated at the Natural History Museum of the University of Cochabamba (in the city of Cochabamba located in the HIGH region), while the sediments from the LOW region were inundated at the Quechuan University “Casimiro Huanca (in the city of Chimoré, located in the LOW region). In both cases, inundation took place outside, in a place sheltered from the sun and the wind. Water used to inundate sediments was similar in pH (range: 7.1–7.2), specific conductance (256–284 μS/cm) and dissolved oxygen saturation. Stream and wetland samples were inundated under identical conditions. Inundation of stream sediments under lentic conditions is a common and successful method to study desiccation‐resistant forms (e.g., Larned et al., 2007; Storey & Quinn, 2013; Stubbington & Datry, 2013).

After 18 days of inundation, the water column of each container was swept vigorously using a 250‐μm hand‐mesh for 30 s to collect invertebrates. The 18‐days inundation period was selected to maximize egg and cyst hatching while avoiding sediment anoxia that could result from longer inundation periods (Larned et al., 2007; Stubbington & Datry, 2013). Water was used to elutriate sediments from each container and poured through a 250‐μm mesh sieve three times (Datry et al., 2012; Larned et al., 2007). Invertebrates collected were preserved in 96% ethanol and further enumerated and identified to the lowest practical level in the laboratory using the Fernandez & Dominguez (2001) and Merritt and Cummins (1996) identification keys. Briefly, insects were identified to the genus level, except Chironomidae, and crustaceans to the genus level except Copepoda and Ostracoda. Hydracarina and Oligochaeta were not identified further (Appendix S1). South American invertebrates are still poorly known, but such coarse resolution was shown to be sufficient to study community patterns across disturbance (e.g., Datry et al., 2014) or large‐scale environmental gradients (e.g., Heino, 2011) and is commonly used for bio‐monitoring in Bolivia (e.g., Datry et al., 2016; Moya et al., 2011).

#### 2.3.2 Invertebrate sampling during the wet period

At each site, three Surber samples (Surber surface area 0.1 m^2^, mesh size 250 μm) were collected from 2 to 3 riffles (streams) and one pond (wetlands) and pooled together to form a 3 L composite sample. While we acknowledge that the use of Surber is not the most appropriate for sampling lentic water bodies due to their lack of flow, using a consistent sampling technique for dry and wet periods and for each ecosystem type was necessary to ensure equal comparisons and thus more robust interpretation of the data. To overcome the lack of flow in ponds, we manually created water movement for 3 min (i.e., to match the time necessary to collect samples in streams) in the front of the Surber to push sediments and organisms into the net. For each sample, sediments were disturbed to a depth of 10 cm to collect organisms from the same sediment volume as those from the dry sediment samples. Invertebrates were preserved, enumerated, and identified as previously described.

### 2.4 Data analysis

Rarefaction curves with 95% confidence intervals were plotted against the number of samples to compare the sampling effort between sediment and benthic communities and streams and wetlands (Gotelli & Colwell, 2001).

For each site across streams and wetlands, we then calculated three different aspects of the contribution of desiccation‐resistant forms to community recovery, accounting for the regional differences in species pool. First, we compared the number of total, shared and unique taxa between dry sediment and benthic communities. The ratio of the number of shared taxa by the total number of taxa in the benthic community defined the contribution of desiccation‐resistant forms to community recovery in terms of shared species. Second, we quantified community dissimilarity between dry sediment and benthic samples for each site, using the Chao index calculated on log‐transformed densities, which is particularly suitable when sample size and taxonomic richness differ among groups (Anderson & Millar, 2004; Cañedo‐Argüelles et al., 2015; Chao, Chazdon, Colwell, & Shen, 2005). The dissimilarity in community composition between dry sediment and benthic communities defined the contribution of desiccation‐resistant forms to community recovery in terms of community similarity. Third, we compared the number of total, shared, and unique functional traits between dry sediment and benthic communities. To do so, twenty functional traits (59 trait states) related to life history, mobility, morphology, ecology, and trophic habitat were assigned to genera or families depending on the level of taxonomic resolution possible (Poff et al., 2006; Tomanova & Usseglio‐Polatera, 2007; Tomanova, Moya, & Oberdorff, 2008; Appendix S1). Although assigning traits at levels coarser than species can reduce the accuracy of trait information (but see Hadly, Spaeth, & Li, 2009), many congeneric species are functionally equivalent (Poff et al., 2006). Genus‐ and family‐level assignment of traits has proven to be sufficient to detect the functional responses of communities to drying in various intermittent systems (e.g., Bonada et al., 2006; Chessman, 2009; Datry et al., 2014, 2016; Leigh & Datry, 2016; Moya et al., 2011; Tomanova et al., 2008; Vander Vorste, Corti, et al., 2016; Vander Vorste, Malard, et al., 2016). Only taxa with sufficient trait data and/or taxonomic resolution were used for functional trait analysis (110 taxa of 187 total identified taxa). Although this excluded some numerically abundant taxa (e.g., Oligochaeta, Chironomidae, Copepoda, and Cladocera, Appendix S1), this missing information was unlikely to bias our results because these taxa were ubiquitous across sampling regions and ecosystem types (frequency of occurrence >85%).

The ratio of the number of shared traits between sample types by the total number of traits in the benthic community defined the contribution of desiccation‐resistant forms to community recovery in terms of functional diversity.

To test our first prediction, we compared the contribution of desiccation‐resistant forms to community recovery as calculated above among LOW, MED, and HIGH regions for streams and between MED and HIGH regions for wetlands using one‐way analyses of variance (ANOVAs) and post hoc Tukey tests. To test our second prediction, we compared the contribution of desiccation‐resistant forms to community recovery as calculated above between wetland and streams for the two regions where both ecosystem types occurred (i.e., HIGH and MED) using one‐way analyses of variance (ANOVAs). In each case, ANOVA models were validated by plotting residuals against fitted values to check for violations of assumed normality and homogeneity.

All statistical analyses were carried out using R software (R Core Team, 2015) and functions in the package Vegan (Oksanen et al., 2013). For all tests, *p *<* *.05 indicated statistical significance.

## 3 Result

### 3.1 Benthic and rewetted sediments communities

**Figure 3:**
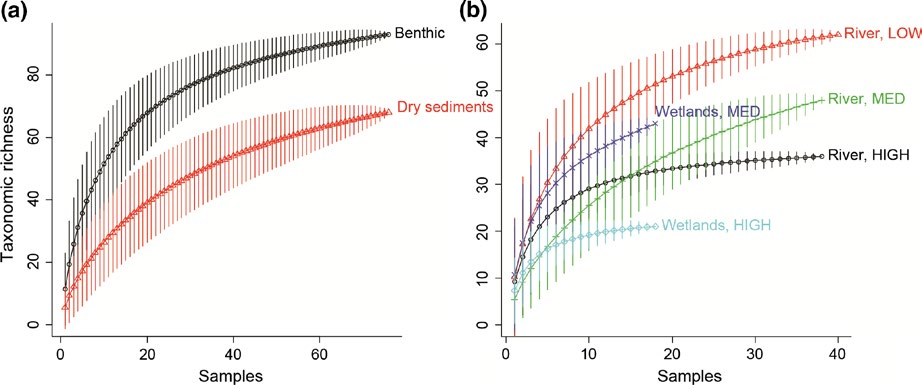
Accumulation curves of taxonomic richness (a) for benthic and dry sediments, (b) for streams and wetlands across low, medium, and high regional drying prevalence regions (LOW, MED, and HIGH, respectively)

Sampling effort was consistent across regions and ecosystem types and accumulation curves indicated it was sufficient to collect most invertebrate taxa and accurately estimate taxonomic richness (Figure 3).

In general, stream communities were more diverse but contained fewer organisms compared to wetland communities (ANOVA *p* < .001, Figure 3 and Appendix S1). From streams, we identified 8,416 invertebrates in benthic sediments belonging to 107 taxa. The most abundant taxa were Chironomidae (Diptera, relative abundance = 58.2%), Oligochaeta (relative abundance = 6.5%), *Meridialaris* sp. (Ephemeroptera, relative abundance = 4.9%), and Neoelmis sp. (Coleoptera, relative abundance = 2.8%). Ninety‐two taxa had relative abundances of <1% and 64 of <0.1%. From wetlands, we identified 14,651 invertebrates in benthic sediments belonging to 46 taxa. The most abundant taxa were Copepoda (relative abundance = 33.8%), Cladocera (relative abundance = 12.9%), Chironomidae (relative abundance = 12.3%), Ostracoda (relative abundance = 12.2%), Hyalella (Amphipoda, relative abundance = 8.1%). 36 taxa had relative abundances of <1% and 23 of <0.1%). Taxonomic richness was highest in the LOW region, lowest in the HIGH region and intermediate in the MED region (Figure 3 and Appendix S1, post hoc Tukey tests, *p* < .01). Density was higher and taxa evenness lower, in the MED region for streams and wetlands (Appendix S1, post hoc Tukey tests, *p* < .01).

Communities from rewetted sediments were similar in terms of taxonomic richness, density and evenness between streams and wetlands (ANOVA, *p* = .452, 0.185, and 0.684, respectively, Appendix S1). From streams, we identified 3,375 invertebrates in rewetted sediments belonging to 82 taxa. The most abundant taxa were Oligochaeta (relative abundance = 40.3%), Chironomidae (Diptera, relative abundance = 20.1%), Hydracarina (relative abundance = 3.7%), and Cladocera (relative abundance = 3.4%). Seventy‐two taxa had relative abundances of <1% and 37 of <0.1%. From wetlands, we identified 1,210 invertebrates in rewetted sediments belonging to 24 taxa. The most abundant taxa were Hydracarina (relative abundance = 28.7%), Chironomidae (relative abundance = 19.7%), Nematoda (relative abundance = 15.4%), and Oligochatea (relative abundance = 13.4%). 13 taxa had relative abundances of <1% and 5 of <0.1%. Taxonomic richness of rewetted sediment communities was highest in the LOW region for streams and lowest in the HIGH region for wetlands (Appendix S1, post hoc Tukey tests, *p* < .05). Density and evenness did not differ among regions for streams (Appendix S1, ANOVA, *p* = .325 and 0.634, respectively) and wetlands (Appendix S1, ANOVA, *p* = .154 and .234, respectively).

### 3.2 Influence of regional drying prevalence

**Figure 4:**
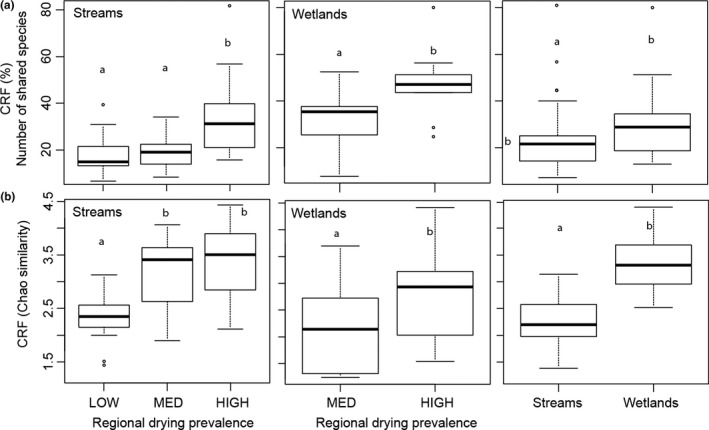
Boxplots (25, 50 and 75 quartiles) showing the contribution of desiccation‐resistant forms to community recovery (CRF, %) in terms of (a) number of species and (b) community composition (Chao similarity) among low, medium, and high regional drying prevalence regions and between streams and wetlands across regions (left panels). Different letters indicate statistical differences

**Figure 5:**
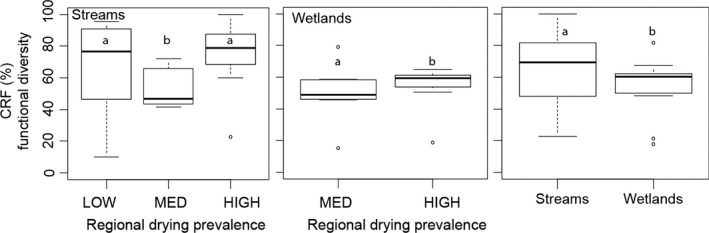
Boxplots (25, 50 and 75 quartiles) showing the contribution of desiccation‐resistant forms to community recovery in terms of functional diversity (CRF, %) across low, medium, and high regional drying prevalence regions and between streams and wetlands across regions (left panels). Different letters indicated statistical differences

In terms of the number of shared taxa, the contribution of desiccation‐resistant forms to community recovery in streams was higher in the HIGH region compared to the MED and LOW regions (Figure 4a, post hoc Tukey tests, *p* = .012 and <.001, respectively). The contribution of desiccation‐resistant forms did not differ significantly between MED and LOW regions (Figure 4a, post hoc Tukey test, *p* = .797). In wetlands, the contribution of desiccation‐resistant forms to community recovery was higher in the HIGH region compared to the MED region (Figure 4a, ANOVA, *p* < .001). With respect to the similarity in community composition, the contribution of desiccation‐resistant forms to community recovery in streams was higher for HIGH and MED region compared to the LOW region (Figure 4b, post hoc Tukey tests, *p* < .001 and .012, respectively). In wetlands, the contribution of desiccation‐resistant forms to community recovery was slightly higher in the HIGH region compared to the MED region (Figure 4a, ANOVA, *p* = .034). The above patterns were similar after removing the two most abundant taxa identified at a coarse taxonomic resolution (i.e., Oligochaeta, Chironomidae for streams and Copepoda and Cladocera for ponds) from each ecosystem type due to their ubiquity across samples (frequency of occurrence >85%). With respect to functional diversity in streams, the contribution of desiccation‐resistant forms to community recovery was higher in the HIGH region than in the MED region (Figure 5, post hoc Tukey HSD, *p* = .008), but did not differ from the LOW region (Figure 5, post hoc Tukey HSD, *p* = .422). With respect to functional diversity in wetlands, the contribution of desiccation‐resistant forms to community recovery was similar between the HIGH and MED regions (Figure 5, ANOVA, *p* = .645).

### 3.3 The influence of ecosystem type

As predicted, in terms of number of shared taxa, the contribution of desiccation‐resistant forms to community recovery was higher in wetlands (mean ± *SD*:28 ± 16%) than in streams (mean ± *SD*:22 ± 9%) (Figure 4a, *p* < .05). With respect to the similarity in community composition, the contribution of desiccation‐resistant forms to community recovery was higher in wetlands (than in streams (Figure 4b, ANOVAs, *p* < .001). In terms of functional diversity, the contribution of desiccation‐resistant forms to community recovery was slightly higher in streams (mean ± *SD*: 67 ± 20%) than in wetlands (mean ± *SD*: 49 ± 16%) (Figure 5, ANOVA *p* = .035), although variability was high for streams.

## 4 Discussion

Shifts from permanent to intermittent water regimes are projected to occur in many areas as a response to global change (e.g., Datry et al., 2014; Döll & Schmied, 2012; Pyne & Poff, 2017). Our results indicate that the subsequent responses of freshwater biodiversity, which is thought to experience the strongest declines in biodiversity of any faunal group (Jenkins, 2003), may be context dependent, varying across regions and ecosystem types. In some regions and freshwater ecosystem types, communities might be more resistant than others and this should be taken into account in projected biodiversity scenarios and mitigation actions. In terms of taxonomic richness, the contribution of desiccation‐resistant forms to community recovery in streams was high (i.e., 33%) in the region where drying commonly occurs across most freshwater habitats, but rather low (i.e., 15%) in the region where freshwater habitats are perennially inundated. Although it was impossible to replicate entire catchments across the different levels of regional drying prevalence due to the challenging access to these regions, our findings are likely driven by contrasted selection of the species with desiccation‐resistant traits from the regional species pool. With high regional drying prevalence, the selection for desiccation‐resistant traits was strongest, whereas the selection was lower in the region with low drying prevalence. The few species with desiccation‐resistant traits occurring in the region with low drying prevalence could simply be the result of selection by random processes from a more diverse species pool (Datry et al., 2016; Tomanova et al., 2008). Average taxonomic richness in this region was 2× higher than in the region with high drying prevalence (Appendix S1). Consequently, expected increases in flow intermittence driven by global change could exacerbate species loss more greatly in regions with normally low drying prevalence.

Lotic and lentic aquatic systems are rarely considered simultaneously when assessing the effect of disturbances, including drying, although cross‐system comparisons are a powerful tool to identify general ecological mechanisms and improve understanding of how biodiversity is altered by global change (Palmer, Allan, & Butman, 1996). Here, we showed that communities in wetlands appear to be more resistant to drying than in streams, regardless of the regional drying prevalence. This is likely due to contrasted hydrologic connectivity, influencing the selective pressure to promote resistance strategies in isolated water bodies. These results will allow refining the predictions of freshwater biodiversity responses to anthropogenic climate change and could help identify key areas or ecosystem types where mitigation efforts should focus.

The contribution of desiccation‐resistant forms to community recovery did not differ between regions of low and medium regional drying prevalence in terms of species richness. This suggests that the selection of species possessing the suitable traits to cope with a given level of disturbance is not always linear along disturbance gradients as reported in different ecosystems (e.g., Bongers, Poorter, Hawthorne, & Sheil, 2009; Imai et al., 2016; Westgate, Driscoll, & Lindenmayer, 2012). There could be a threshold in disturbance levels that promotes the selection of species with the suitable traits, but testing this hypothesis will require additional data from many regions. Conversely, at low levels of regional drying prevalence, the role of other environmental factors not taken into account in this study (e.g., predator presence, flood regime) might have increasing influence on communities.

Positive cotolerance occurs when traits that enhance resistance and resilience to one type of disturbance also increase tolerance to other disturbances (Côté & Darling, 2010; Vinebrooke et al., 2004). It could explain why the contribution of desiccation‐resistant forms to community recovery in streams did not differ between moderate and high drying prevalence regions and why the differences between moderate and high drying prevalence regions for wetlands were not as strong when using community similarity instead of the number of species shared between rewetted sediments and benthic samples. In the Sajama region, although the regional drying prevalence is lower than in the Cochabamba region due to the presence of many glacier‐fed streams, streams are also prone to frequent freezing due to very low nocturnal temperatures (Moya et al., 2009). Although poorly investigated, drying and freezing could favor the selection of traits, notably those related to physiological tolerance, that enable species to survive both disturbances. Such positive cotolerance could explain the high contribution of desiccation‐resistant forms to community recovery to community composition observed in the moderate and high drying prevalence regions. Although not often considered by freshwater ecologists (Vander Vorste, Corti, et al., 2016), the varying responses of freshwater communities to drying (Bogan et al., 2013; Datry et al., 2014) could be partially attributable to positive cotolerance with other disturbances, such as freezing. Further investigating the effects of drying on community resilience across glacial streams represents a promising research avenue to explore the potential influence of cotolerance processes.

The contribution of desiccation‐resistant forms in streams and wetlands seemed to contribute greatly to functional diversity. The high proportion of functional traits in streams (mean = 67%) and wetlands (mean = 49%) present in taxa surviving using desiccation‐resistant forms suggests that desiccation‐resistance contributes to ecosystem function by buffering communities from the loss of functional traits (Rosenfeld, 2002). Our dataset was likely limited by the coarse taxonomic resolution inherent to poorly explored biogeographical regions, including trait information from taxonomic groups that respond to flow intermittence gradients (e.g., Chironomidae, Cañedo‐Argüelles, Bogan, Lytle, & Prat, 2016) would likely strengthen our findings of the importance of desiccation‐resistant forms to functional diversity. Considering that river and wetland invertebrate communities influence multiple ecosystem functions (e.g., primary productivity, organic matter decomposition), the recovery of functional diversity may be as important as taxonomic richness to overall ecosystem resilience (Gallagher, Hughes, & Leishman, 2013; Mouillot, Villéger, Scherer‐Lorenzen, & Mason, 2011). These results highlight that the production of desiccation‐resistant forms can be an important mechanism that promotes the recovery of ecosystem function in the face of increased disturbances caused by climate change. This is corroborated by an increasing number of studies reporting high resistance of functional diversity in freshwater ecosystems (Leigh et al., 2016; Schriever et al., 2015; Vander Vorste, Corti, et al., 2016; Vander Vorste, Malard, et al., 2016).

Our study is the first to show the influence of regional drying patterns on community recovery in lotic and lentic aquatic temporary ecosystems. Similar context dependency in the variability of zooplankton hatching dynamics was recently explained in Western Canada across a 1,800‐km‐long latitudinal gradient (Jones & Gilbert, 2016). The authors showed that cues associated with climate can have consistent (phenology) or distinct (abundance, richness) effects at different latitudes, hindering any prediction of the effect of climate change on community recovery. Although the contribution of desiccation‐resistant forms to community recovery was not assessed in the aforementioned study, the systematic differences in hatching reported along the latitudinal gradient might cascade onto community dynamics. While the physiological aspects of desiccation resistance are well known (e.g., Bewley, 1979), more research is needed to understand its significance for population and community resilience, especially in a context of global change. Exploring the ecological consequences of temporal dispersal through desiccation resistance in dry sediments in temporary freshwaters could help improve our ability to predict the effects of global change, notably an increase in drying prevalence, across broad geographic areas.

As expected, the contribution of desiccation‐resistant forms to community recovery was higher in wetlands than in streams regardless of regional drying prevalence. This held for the number of species shared between wet and dry hydrologic phases and in terms of community similarity based on abundance. In streams, where hydrologic connectivity is high because flow connects entire river network, at least during wet phases (Datry et al., 2016), evolutionary cues to develop desiccation‐resistant strategies might not be as strong as when connectivity is low or absent, such as among disconnected wetlands. In these systems, dispersal is restricted to species with aerial life stages or passively by mechanisms such as zoochory (Bohonak & Jenkins, 2003). In the isolated wetlands studied here, as in other temporary lentic waters (e.g., vernal pools), evolution might have favored the development of resistance strategies (Collinge & Ray, 2009; Rice & Emery, 2003). Contrarily, in all streams studied here, perennial refuges (e.g., perennial reaches, springs) occurred upstream, promoting the recovery of downstream communities through passive and active drift (Bohonak & Jenkins, 2003). More cross‐system comparisons (i.e., lotic vs. lentic) of the strategies used by organisms to persist along disturbance gradients may help disentangle the role of microevolution in shaping biodiversity, notably in aquatic, temporary systems. Accurately predicting the responses of freshwater biodiversity to global change including increased drying will necessitate accounting for context dependency of the mechanisms that promote the resistance and resilience of communities.

## Conflict of Interest

None declared.

## Data Accessibility

The fauna dataset will be deposited on the Dryad Digital Repository (www.http://datadryad.org/) upon acceptance of the manuscript.

## Author Contributions

TD initiated the study, designed, and carried out the experiment and data analyses and wrote the MS. RVV contributed to the data analyses and to the MS, TO, and MC contributed to the study design and TO contributed to the MS. NM, JZ, EG, and FR participated to the experiments and carried out sample analyses.
